# Whether Keratectasia Area Shown in Corneal Topography Is Appropriate for Evaluating the Effect of Corneal Cross-Linking for Keratoconus: A 12-Month Follow-Up Study

**DOI:** 10.1155/2019/1762537

**Published:** 2019-04-03

**Authors:** Jia Wang, Zhiwei Li, Huankai Zhang, Ning Gao, Guoying Mu

**Affiliations:** ^1^Department of Ophthalmology, Shandong Provincial Hospital Affiliated to Shandong University, Jinan 250021, China; ^2^Department of Ophthalmology, The Second People's Hospital of Liaocheng, Linqing 252600, China

## Abstract

**Purpose:**

To analyze the keratectasia area (KEA) shown in corneal topography before and after corneal cross-linking (CXL) in patients with progressive keratoconus (KC) and figure out whether KEA is appropriate for evaluating the effect of CXL.

**Methods:**

A retrospective analysis was conducted in 34 eyes from 24 progressive KC patients who have underwent CXL from 2015 to 2017. Area with* K-*value more than 47D shown in the corneal topography was marked and identified as KEA. Keratometry (*K*1,* K*2, and* K*max), KEA, thinnest corneal thickness (TCT), and endothelial cell density (ECD) were evaluated preoperatively or at months 3, 6, and 12 postoperatively. The changes of KEA before and after operation were evaluated. The relation of KEA and other parameters, including* K*max and TCT, was analyzed.

**Results:**

Linear regression model revealed the KEA,* K*max,* K*1, and* K*2 decreased after CXL in model y = 0.9622 -0.02408 x (P<0.05), y = 0.9982 -0.003469 x(P<0.05), y = 0.9977 + -0.001347 x(P<0.05), y = 0.9992 + -0.001779 x(P<0.05) (y represents KEA,* K*max,* K*1, or* K*2; x represents time (month)). The KEA is significantly decreased in early stage (before month 3) (P<0.05); however, the* K*max,* K*1, and* K*2 have no significant decrease in early stage (P= 0.09, 0.19, 0.32).

**Conclusions:**

The KEA is more sensitive than* K-*value in describing the morphological changes of cornea after CXL, especially in early stage after treatment.

## 1. Introduction

Keratoconus (KC) is a bilateral, noninflammatory disease characterized by a cone-shaped protrusion on the anterior corneal surface, which results in corneal thinning, progressive myopia, irregular astigmatism, corneal scaring, and significant visual impairment [1]. According to recent literature, the prevalence and annual incidence of KC are 1:375 and 1:7,500, respectively, which are five- to tenfold higher than those in previous report [2]. Although many established therapies, such as rigid gas-permeable contact lenses and corneal transplantation, are effective in improving KC patient vision, corneal cross-linking (CXL) was the only minimally invasive treatment to improve corneal biomechanical property and halt the progression of KC [3–5].

During the postoperative follow-up, corneal topography has been considered as an indispensable assistant tool for evaluating the CXL effect. Different degrees of* K-*value decrease were recorded in keratoconic patients after CXL [6]. In the clinic, we found that the changes of keratectasia area (KEA) are sensitive to the variation of* K*-value and other KC related parameters, which merits a study to evaluate the relation of KEA and the KC situation.

Herein we retrospectively evaluate the changes of KEA in KC patients who received CXL, and explore the clinical significance of KEA in evaluating the effect of CXL for KC. In this study, we defined the region above 47D in corneal topography as KEA based on diagnostic criteria of Rabinowtz for KC [7, 8].

## 2. Patients and Methods

### 2.1. Patients

Present study involved a total of 34 eyes from 24 patients (15 males, 9 females; mean age: 22.88 ± 6.40 years, range: 11-35 years) diagnosed with progressive keratoconus and underwent CXL between July 2015 and July 2017 at the Department of Ophthalmology, Shandong Provincial Hospital affiliated to Shandong University. Patients with 1 or more of following signs over 2 years were identified as progressive keratoconus: an increase of more than 1.00 diopter (D) in* K*max based on the placido corneal topography; an increase of more than 1.00 (D) in cylinder; and an increase of more than 0.50 D in spherical equivalent [9].

Exclusive criteria included a history of herpes simplex keratitis, corneal surgery or chemical injury, or corneal topography not well structured for calculation of KEA.

This study was approved by the ethics committee of Shandong Provincial Hospital affiliated to Shandong University, and written informed consent was obtained from all the patients before study initiation.

Corneal topography (Allegro Topolyzer Vario; Wave Light GmbH, Erlangen, Germany), central corneal thickness (CCT) (OCT, Cirrus HD-OCT 4000; Carl Zeiss Meditec Inc., Hacienda Drive, Dublin, CA), and corneal endothelial cell density (ECD) (Specular Microscope SP-3000P; Topcon Corporation, Tokyo, Japan) were evaluated before treatment. Corneal topography was also obtained at each follow-up time to evaluate the effect of CXL treatment. All patients were followed up for at least 1 year.

### 2.2. Surgical Technique

CXL was performed according to the conventional Dresden protocol described by Wollensak et al. [10] and Xu et al. [11]. All operations were performed by the same surgeon under sterile conditions. The corneal epithelium within the central 9 mm diameter area was removed mechanically after topical anesthesia using proparacaine hydrochloride 0.5% eye drops (Alcaine; Alcon Laboratories, Inc., Fort Worth, TX). Subsequently, denuded stroma was administered topically with 0.1% isotonic solution of riboflavin (TCT>400* μ*m) or 0.1% hypoosmolar solution of riboflavin (TCT<400* μ*m) every 3 minutes for 30 minutes.

0.1% riboflavin was generated by diluting 0.5% riboflavin solution (Shandong Fangming Pharmaceutical Limited by Share Ltd, Shandong, China) with physiological salt solution (sodium chloride 0.9% solution; 310 mOsmol/L; Sichuan Kelun Pharmaceutical Limited by Share Ltd, Sichuan, China) or sterile water. In order to avoid damage of ultraviolet A (UV-A) irradiation to endothelium, lens, and deeper structures [12, 13], hypoosmolar riboflavin solution was applied in patients with TCT less than 400* μ*m to ensure that the thinnest stroma swollen to 400* μ*m or more measured by optical coherence tomography (OCT, Cirrus HD-OCT 4000; Carl Zeiss Meditec Inc, Hacienda Drive, Dublin, USA).

After confirming the presence of riboflavin in the anterior chamber using Slit lamp with a blue filter, we irradiated the cornea with ultraviolet A (370 nm, 3.0 mW/cm^2^, UV-X illumination system version 1000, UVXTM; IROCAG, Zurich, Switzerland) with a 5 cm working distance for 30 minutes, with continued application of riboflavin drops every 5 minutes.

The cornea was immediately rinsed with physiological salt solution following CXL. A bandage contact lens was placed after CXL. The bandage contact lens was not removed until corneal re-epithelialization on day 3 to day 5. Postoperative medication included 0.5% levofloxacin eyedrops (Cravit; Santen Pharmaceutical Company, Osaka, Japan) 4 times daily for 1 week and 0.1% fluorometholone (Fluorometholone Eye Drops; Allergan Pharmaceuticals Ireland, Westport, County Mayo, Ireland) 4 times daily for 2 weeks.

### 2.3. Area Measurement

KEA was identified as area with* K*-value above 47D shown in the tangential corneal topography. KEA was measured with Image Pro Plus 6.0 (IPP) software by setting the area of interest (AOI) according to relative color scale and the annotation of* K*-value which was displayed after the mouse click on the corneal tangential map. The KEA was measured using IPP based on the inbuilt scale length of the corneal topography. The AOI has to be traced manually.

### 2.4. Statistical Analysis

*K*-value or KEA ratio was obtained through dividing the data in specific time-point with the data before operation. Statistical analysis was performed using SPSS software version 20.0 (IBM, Armonk, NY, USA). Data were recorded as mean ± standard deviation. Pearson correlation, linear regression, or ANOVA was used when needed. P<0.05 was considered significant.

## 3. Results

Corneal epithelium recovered well and no serious complications such as infection or corneal scar were observed after treatment in all patients.

**Figure 1:**
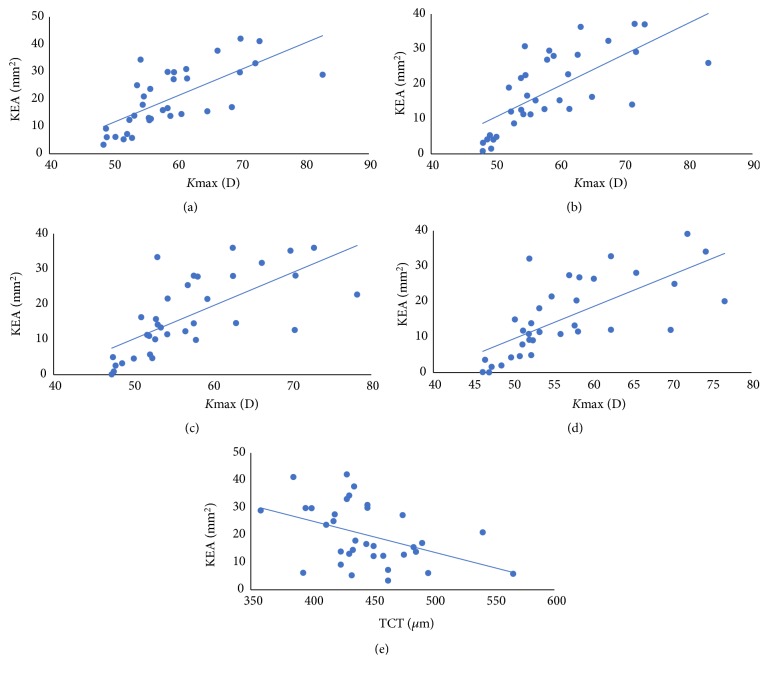
The scatter plot between KEA and* K*max preoperatively and at months 3, 6, and 12 (a-d). The scatter plot between KEA and TCT preoperatively (e).

There were strong positive correlations between KEA and* K*max preoperatively and at months 3, 6, and 12 postoperatively (r=0.762, 0.778, 0.775, 0.777; P<0.05). There was a medium negative correlation between KEA and TCT preoperatively (r=-0.430, P<0.05) (Figure 1).

The mean* K*max,* K*1,* K*2, and KEA were 58.89 D (SD±7.97), 46.68 D (SD±3.80), and 50.12 D (SD±5.07), 20.06 mm^2^ (SD ±11.03) preoperatively; 58.36 D (SD±8.50), 46.37 D (SD±4.31), and 49.83 D (SD±5.90), 18.00 mm^2^ (SD±10.91) at month 3; 57.14 D (SD±8.01), 46.06 D (SD±4.33), and 49.57 D (SD±5.79), 16.78 mm^2^ (SD±10.95) at month 6; and 56.55 D (SD±8.25), 45.92 D (SD±4.15), and 49.06 D (SD±5.46), 15.36 mm^2^ (SD±10.75) at month 12 (Table 1).

**Figure 2:**
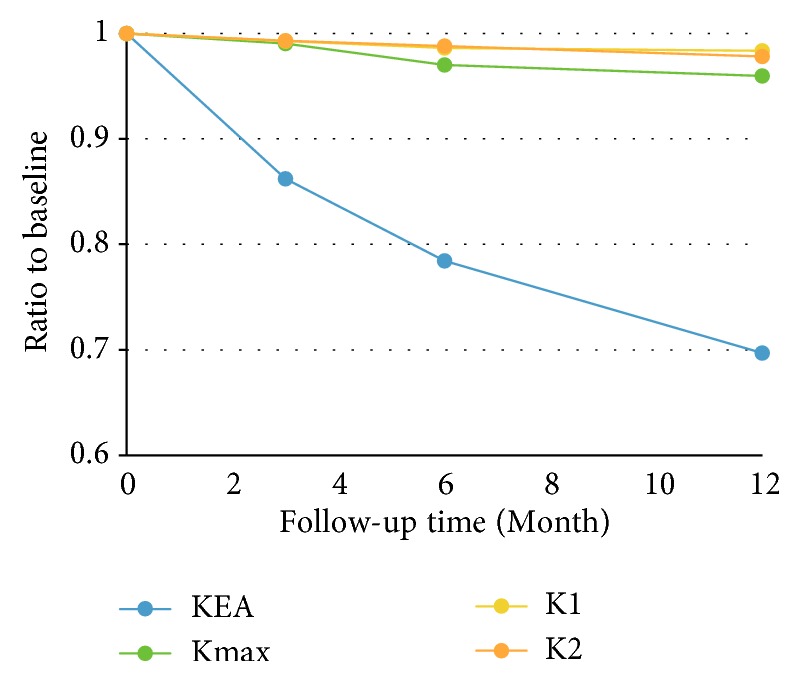
Linear regression model revealed the KEA,* K*max*, K*1, and* K*2 decreased after CXL in model y = 0.9622 -0.02408 x (P<0.05), y = 0.9982 -0.003469 x(P<0.05), y = 0.9977 + -0.001347 x(P<0.05), y = 0.9992 + -0.001779 x(P<0.05) (y represents KEA,* K*max*, K*1 or* K*2; x represents time (month)).

Linear regression model revealed the KEA,* K*max,* K*1, and* K*2 decreased after CXL in model y = 0.9622 -0.02408 x (P<0.05), y = 0.9982 -0.003469 x(P<0.05), y = 0.9977 + -0.001347 x(P<0.05), y = 0.9992 + -0.001779 x(P<0.05) (y represents KEA,* K*max,* K*1 or* K*2; x represents time (month)) (Figure 2). Although all of the parameters were positive related to follow-up time-point, the slope of KEA decrease is 10-20-fold larger than that of* K*-values, which implies that the KEA is much more sensitive to the cornea morphology variation secondary to CXL.

Result of ANOVA combined Turkey analysis substantiated the result of regression analysis. In detail, the KEA was significantly decreased in early stage (before month 3) (P<0.05); however, the* K*max,* K*1, and* K*2 had no significant decrease in early stage (P= 0.09, 0.19, 0.32).

TCT and ECD did not show statistically significant variation pre- and postoperatively during the follow-up periods (P>0.05).

## 4. Discussion

In 2003, Wollensak et al. firstly reported the CXL induced by riboflavin/ultraviolet A for the treatment of KC [10]. Since then, several similar studies have been conducted around the world and confirmed its long-term safety and efficacy in halting the progression of KC [14–17]. Many new protocols have been proposed, such as transepithelial CXL and accelerated CXL. Although there are fewer complications or shorter UVA irradiation time, these modified techniques are less effective, particularly in stabilizing or decreasing* K*max [2, 18]. Up to now, the conventional Dresden protocol utilized in this study is still the main technology for KC treatment.

Corneal topography is a widespread clinical practice for evaluating the efficacy of CXL treatment. The main use of corneal topography is the generation of indices that allows quantifying the level of irregularity of the corneal morphology, at a local or general level [19]. In the present study, we used IPP software to measure KEA region in which keratometry value was above 47D. We choose tangential map with relative color scale as a resource to measure KEA because this map represents a high sensitivity to data obtained [19] and relative color scale is sensitive to small changes [20].

In our results, the KEA decreased from the baseline of 20.06±11.03 mm^2^ to 18.00±10.91 mm^2^, 16.78±10.95 mm^2^, and 15.36±10.75 mm^2^ at months 3, 6, and 12 postoperatively, respectively. The reduction of KEA may be due to the flatting effect of CXL by arousing additional covalent bonds between and within collagen fibrils of the cornea taking advantage of photo-oxidation reaction [2, 10], which has been demonstrated in several articles [21–24].

*K*-values, especially* K*max, were essential indices for evaluating the efficacy of CXL. Recently, different degrees of improvement in* K*max were recorded within a one-year follow-up after CXL. Uysal et al. [25] reported that 109 eyes had a stable* K*max (45.9%) or a decreased* K*max up to 3.0 D (52.2%) one year after CXL treatment based on a retrospective study with 111 eyes recruited. Kumar et al. [26] reported that the preoperative* K*max was 55.11 D (SD±5.34), whereas the mean of postoperative* K*max was reduced to 53.87 D (SD±4.99) based on their retrospective study recruiting 34 eyes. The present study revealed* K*max decreased from 58.89 (SD±7.97D) before CXL to 56.55 (SD±8.25D) at one year after CXL. We also found significant reductions of* K*1 and* K*2 at months 6 and 12 after CXL treatment. A positive correlation was found between KEA and* K*max before or after CXL (Figure 1).

We also found significant medium negative correlation between KEA and TCT preoperative (Figure 1). TCT is one of important signs of KC progression [27] and TCT in a suspect KC is significantly lower than normal eyes and higher than the KC group [28]. Therefore, the more serious KC led to the lower TCT and larger KEA. We suspect that the KEA may be beneficial in monitoring KC as TCT.

The changes in KEA and* K*-value compared to preoperative at each follow-up were all increasingly apparent after CXL in this study (Table 1). The changes of KEA were much more pronounced than that of* K*-value (Figure 2), and changes of KEA but not* K*-value were found within 3 months after CXL, which implies KEA is a more sensitive parameter to reflect the morphology changes of cornea.

In conclusion, we found that the KEA is more sensitive than* K*-value in describing the morphological changes of cornea after CXL in this study, especially in early stage after treatment. Present conclusion merits a further investigation on evaluating the significance of KEA in the diagnosis of forme fruste KC or KC in very early stage. However, measuring KEA using IPP software manually in the corneal topography was somewhat tedious and we hope to find a more convenient measurement method. Moreover, larger sample sizes and further long-term follow-up studies are needed to confirm our results.

## Figures and Tables

**Table 1:** Baseline and 3-, 6-, and 12-month outcomes after CXL in all eyes (n =34).

Parameter	Preop	3 mo postop	6 mo postop	12 mo postop
*K*max (D)	58.89±7.97	58.36±8.50	57.14±8.01	56.55±8.25
*K*1(D)	46.68±3.80	46.37±4.31	46.06±4.33	45.92±4.15
*K*2(D)	50.12±5.07	49.83±5.90	49.57±5.79	49.06±5.46
KEA(mm^2^)	20.06±11.03	18.00±10.91	16.78±10.95	15.36 ± 10.75

*K*1, flattest keratometry reading; *K*2, steepest keratometry reading; *K*max, maximum keratometry; KEA, area of the region above 47 D shown in corneal topography.

## Data Availability

The data used to support the findings of this study are included within the article.

## References

[1] Vazirani J., Basu S. (2013). Keratoconus: Current perspectives. *Clinical Ophthalmology*.

[2] Shalchi Z., Wang X., Nanavaty M. A. (2015). Safety and efficacy of epithelium removal and transepithelial corneal collagen crosslinking for keratoconus. *Eye*.

[3] Jouve L., Borderie V., Sandali O. (2017). Conventional and iontophoresis corneal cross-linking for keratoconus: efficacy and assessment by optical coherence tomography and confocal microscopy. *Cornea*.

[4] Labate C., De Santo M. P., Lombardo G., Lombardo M. (2015). Understanding of the viscoelastic response of the human corneal stroma induced by riboflavin/UV-A cross-linking at the nano level. *PLoS ONE*.

[5] Abbondanza M., Abbondanza G., De Felice V. (2017). Mini asymmetric radial keratotomy and corneal cross-linking for the treatment of a bilateral stage IV keratoconus in a 14-year-old child. *Medical Archives*.

[6] Subasinghe S. K., Ogbuehi K. C., Dias G. J. (2018). Current perspectives on corneal collagen crosslinking (CXL). *Graefe's Archive for Clinical and Experimental Ophthalmology*.

[7] Rabinowitz Y. S. (1993). Corneal topography. *Current Opinion in Ophthalmology*.

[8] Rabinowitz Y. S. (1998). Keratoconus. *Survey of Ophthalmology*.

[9] Hersh P. S., Greenstein S. A., Fry K. L. (2011). Corneal collagen crosslinking for keratoconus and corneal ectasia: one-year results. *Journal of Cataract & Refractive Surgery*.

[10] Wollensak G., Spoerl E., Seiler T. (2003). Riboflavin/ultraviolet-a-induced collagen crosslinking for the treatment of keratoconus. *American Journal of Ophthalmology*.

[11] Xu L., Tao X., Li Z. (2018). Clinical study of mitomycin C in reducing haze formation after ultraviolet a/riboflavin crosslinking for keratoconus. *Eye & Contact Lens-science & Clinical Practice*.

[12] Wollensak G., Spoerl E., Wilsch M., Seiler T. (2003). Endothelial cell damage after riboflavin-ultraviolet—a treatment in the rabbit. *Journal of Cataract & Refractive Surgery*.

[13] Wollensak G., Spoerl E., Wilsch M., Seiler T. (2004). Keratocyte apoptosis after corneal collagen cross-linking using riboflavin/UVA treatment. *Cornea*.

[14] Kymionis G. D., Grentzelos M. A., Liakopoulos D. A. (2014). Long-term follow-up of corneal collagen cross-linking for keratoconus—the cretan study. *Cornea*.

[15] O'Brart D. P. S., Kwong T. Q., Patel P., McDonald R. J., O'Brart N. A. (2013). Long-term follow-up of riboflavin/ultraviolet A (370 nm) corneal collagen cross-linking to halt the progression of keratoconus. *British Journal of Ophthalmology*.

[16] Raiskup F., Theuring A., Pillunat L. E., Spoerl E. (2015). Corneal collagen crosslinking with riboflavin and ultraviolet-a light in progressive keratoconus: ten-year results. *Journal of Cataract & Refractive Surgery*.

[17] O'Brart D. P. S., Patel P., Lascaratos G. (2015). Corneal cross-linking to halt the progression of keratoconus and corneal ectasia: seven-year follow-up. *American Journal of Ophthalmology*.

[18] Shajari M., Kolb C. M., Agha B. (2018). Comparison of standard and accelerated corneal cross-linking for the treatment of keratoconus: A meta-analysis. *Acta Ophthalmologica*.

[19] Cavasmartínez F., De l. C. S. E., Nieto M. J. (2016). Corneal topography in keratoconus: state of the art. *Eye & Vision*.

[20] Piñero D. P., Nieto J. C., Lopez-Miguel A. (2012). Characterization of corneal structure in keratoconus. *Journal of Cataract & Refractive Surgery*.

[21] Herber R., Kunert K. S., Veliká V., Spoerl E., Pillunat L. E., Raiskup F. (2018). Influence of the beam profile crosslinking setting on changes in corneal topography and tomography in progressive keratoconus: Preliminary results. *Journal of Cataract & Refractive Surgery*.

[22] Males J. J., Viswanathan D. (2018). Comparative study of long-term outcomes of accelerated and conventional collagen crosslinking for progressive keratoconus. *Eye (Basingstoke)*.

[23] Han Y., Xu Y., Zhu W. (2017). Thinner corneas appear to have more striking effects of corneal collagen crosslinking in patients with progressive keratoconus. *Journal of Ophthalmology*.

[24] Koller T., Pajic B., Vinciguerra P., Seiler T. (2011). Flattening of the cornea after collagen crosslinking for keratoconus. *Journal of Cataract & Refractive Surgery*.

[25] Uysal B. S., Sarac O., Yaman D., Akcay E., Cagil N. (2018). Optical performance of the cornea one year following keratoconus treatment with corneal collagen cross-linking. *Current Eye Research*.

[26] Kodavoor S. K., Arsiwala A. Z., Ramamurthy D. (2014). One-year clinical study on efficacy of corneal cross-linking in Indian children with progressive keratoconus. *Cornea*.

[27] Safarzadeh M., Nasiri N. (2016). Anterior segment characteristics in normal and keratoconus eyes evaluated with a combined Scheimpflug/Placido corneal imaging device. *Journal of Current Ophthalmology*.

[28] Piñero D. P., Alió J. L., Alesón A., Vergara M. E., Miranda M. (2010). Corneal volume, pachymetry, and correlation of anterior and posterior corneal shape in subclinical and different stages of clinical keratoconus. *Journal of Cataract & Refractive Surgery*.

